# Supplementary figures and images for: A New Probiotic Formulation Promotes Resolution of Inflammation in a Crohn’s Disease Mouse Model by Inducing Apoptosis in Mucosal Innate Immune Cells
Source: Int J Mol Sci. 2024 Nov 10;25(22):12066. doi: 10.3390/ijms252212066 (PMC11593709; doi:10.3390/ijms252212066)

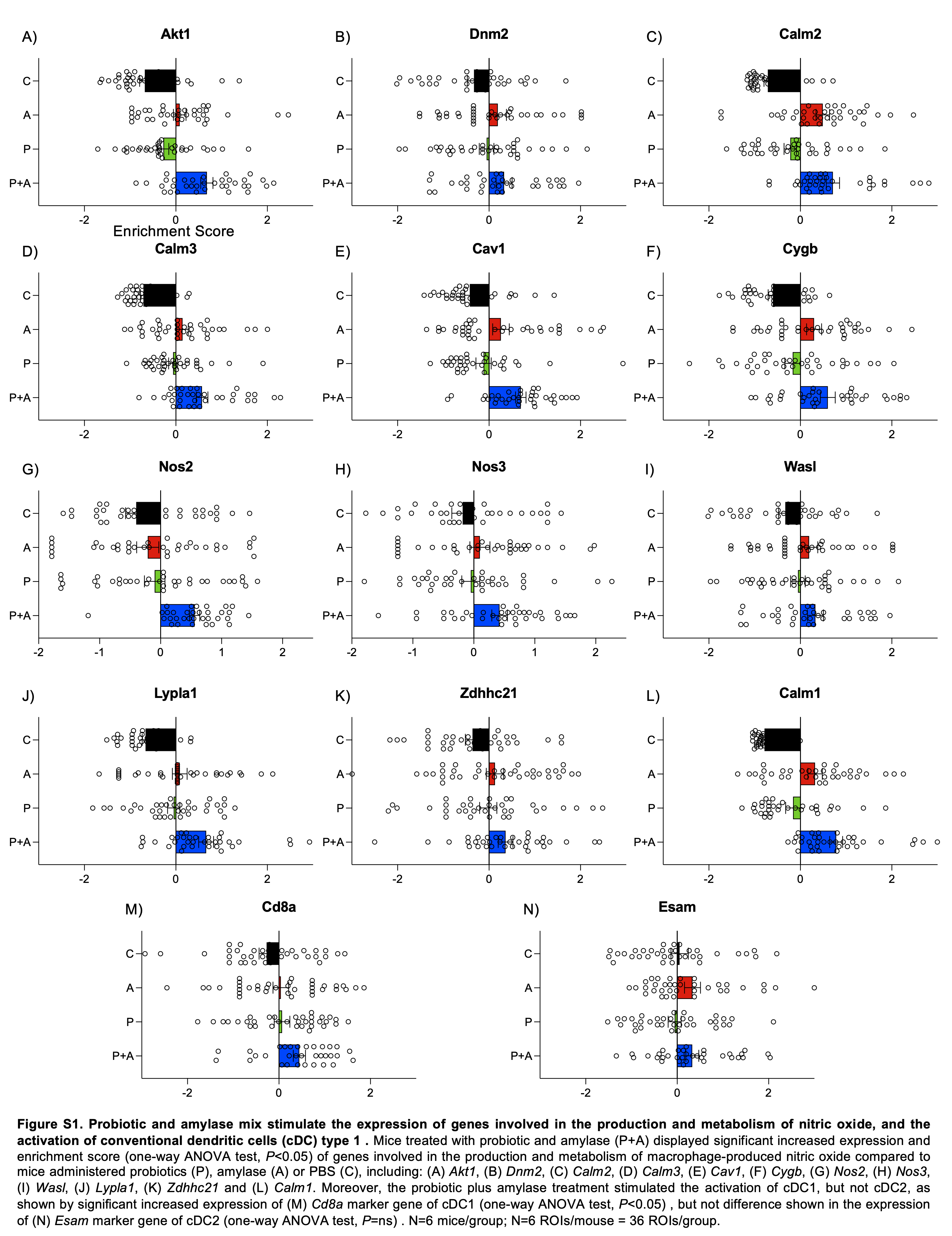

Supplement: Supplementary file 1 [file ijms-25-12066-s001.zip › Figure S1 NOVEMBER 3RD 2024.tiff]
